# Chloral Hydrate

**Published:** 1878-03-20

**Authors:** J. A. Larrabee

**Affiliations:** Professor of Diseases of Women and Children, Hospital Medical College


### CHLORAL HYDRATE.

### A Local Antiseptic and Disinfectant in Puerperal Diseases.

By J. A. Larrabee,
Professor of Diseases of Women and Children, Hospital Medical College.

Widely different views are entertained concerning the contagiousness of so-called puerperal fever. Not more united are the ideas advanced in regard to treatment. Great medical talent and acknowledged skill have been arraigned upon either side. In regard to the contagious or infectious nature of the disease, these opposing opinions come alike from men whose eminence in the profession and in society entitles them to respect. An exhaustive paper, then, upon this subject would simply contain that which should be found in every doctor's library. The one great thing in which the community, no less than the physician, is interested, is our ability to prevent puerperal peritonitis, or stay its progress.

I have several times called the attention of the profession to the disinfectant, deodorizing, and antiseptic properties of chloral hydrate. (See "Virginia Medical Monthly and Transactions of Medico-Chirurgical Society.) Recently, however, I have had an opportunity to observe these properties exerted under most unfavorable circumstances. At the time I took medical charge of the Forest Hill Lying-in Hospital, there had been four deaths, in a period of a few days, from puerperal fever, being, as I believe, peritonitis of the septic form. Everything was in disorder and confusion. Carbolic acid perfumed the place; it was used for everything—lotions, washes, etc.—under the prevalent but mistaken idea of its antiseptic properties. I immediately substituted chloral water, directing that in each delivery it should be well used. Thirty-nine deliveries have been accomplished since that time without any untoward result. The immediate effects of the irrigation or lotion are described by the patients as cooling and pleasant.

In all accouchments, whether liable to contagion from without or not, there exist the necessary conditions within the uterus and vagina for setting up septic poisoning *de novo.* There is, in all cases, an odor to the lochia plainly discernible at the end of the first twenty-four hours. If this condition remains uncorrected, and the nurse neglects to attend to her duties, there is great danger of septic poisoning. A solution of chloral of mild strength in water, and by means of the douche or fountain syringe, removes at once, not only the odor, but, I am fully satisfied, destroys the noxious influence of such poison. Carbolic acid, although it has been much lauded, is, in my judgment, over-estimated, is entirely unreliable, and merely substitutes its own odor for that of the disease.—*Med. News.*

